# Supplementary material for: Unlike its Paralog LEDGF/p75, HRP-2 Is Dispensable for MLL-R Leukemogenesis but Important for Leukemic Cell Survival
Source: Cells. 2021 Jan 19;10(1):192. doi: 10.3390/cells10010192 (PMC7835958; doi:10.3390/cells10010192)
Supplement: Supplementary file 1 [file cells-10-00192-s001.pdf]

Supplementary information

Unlike its paralog LEDGF/p75, HRP-2 is dispensable for MLL-r leukemogenesis but important for leukemic cell survival

Siska Van Belle[1,a], Sara El Ashkar[1,a], Katerina Cermáková[2], Filip Matthijssens[3,4], Steven Goossens[4,5], Alessandro Canella[1], Courtney H. Hodges[6], Frauke Christ[1], Jan De Rijck[1], Pieter Van Vlierberghe[3,4], Václav Veverka[2,7] and Zeger Debyser[1,*]

[1] Laboratory for Molecular Virology and Gene Therapy, Department of Pharmaceutical and Pharmacological Sciences, KU Leuven, Leuven, Flanders, Belgium;
[2] Institute of Organic Chemistry and Biochemistry, Academy of Sciences of the Czech Republic, Flemingovo nam. 2, Prague 6, 166 10 Czech Republic;
[3] Department of Biomolecular Medicine, Ghent University, Ghent, Belgium;
[4] Cancer Research Institute Ghent (CRIG), Ghent, Belgium;
[5] Department of Diagnostic Sciences, Ghent University, Ghent, Belgium;
[6] Department of Molecular & Cellular Biology, Center for Precision Environmental Health, and Dan L Duncan Comprehensive Cancer Center, Baylor College of Medicine, Houston, TX 77030, USA;
[7] Department of Cell Biology, Faculty of Science, Charles University, Prague, Czech Republic.
[a] These authors contributed equally to the work.

* Correspondence: Zeger.debyser@kuleuven.be; Tel.: +32 16 37 40 29

Materials and Methods

Plasmids. The Murine Stem Cell Virus (MSCV) retroviral expression vectors pMSCV-MLL-ENL-Neo and pMSCV-Neo were provided by Prof. Akihiko Yokoyama (Laboratory for Malignancy Control Research, Kyoto University, Japan. The pMSCV retroviral expression vector encoding MLL-AF9 fusion or eGFP (pMSCV IRES-eGFP-PGK-Puro) and the pMSCV-E2A-HLF-Neo were provided by Prof. Jürg Schwaller (Laboratory of childhood leukemia, Basel University, Switzerland). The lentiviral vector expressing mi30-based RNA targeting HRP-2 or eGFP control and retroviral vectors expressing Ledgf/p75 miRNA were described earlier [1,2]. Point mutations blocking the MLL-MENIN interaction (F9A, P10A and P13A) were introduced in the p3xFlag-MLL_{1-330}-ELL construct by oligonucleotide annealing. For NMR experiments, the expression plasmid coding for LEDGF/$p75_{345-426}$, was used as described earlier [3]. The expression plasmid for HRP-$2_{469-549}$ was created by amplification of the HRP-2 fragment from pMAL-HRP-2 [1] and inserted into a pMCSG7 plasmid modified to encode an N-terminal His6 affinity tag followed by the tobacco etch virus (TEV) protease recognition site [4] using NheI/BamHI restriction sites. Upon TEV cleavage, the encoded protein retains a cloning artefact of 5 amino acid residues (SNAAS) at the N-terminus and 2 amino acid residues (GS) at the C-terminus. The integrity of all plasmids was verified by DNA sequencing. All restriction enzymes were purchased from Thermo scientific (Belgium).

Antibodies. Proteins were detected on western blot using specific antibodies against LEDGF/p75 (A300-848A, Bethyl Laboratories), HRP-2 (15134-1-AP, Proteintech Europe), Menin (A300-105A, Bethyl Laboratories), HA (ab215069, Abcam), Flag (F7425, Sigma) or GAPDH (ab9485, Abcam)

Isolation and culture of mouse progenitor cells. Lineage depleted (lin-) progenitor cells were isolated using the EasySep Mouse Hematopoietic Progenitor Cell Isolation Kit (STEMCELL Technologies) on red blood cell lysed bone marrow cells harvested from femur and tibia from 6 to 8-week-old mice. Cells were cultured in RPMI-1640 (10% fetal calf serum, 50 µg/mL gentamicin), supplemented with murine interleukin-6 (10 ng/mL, PeproTech), murine interleukin-3 (6 ng/mL, PeproTech), and murine stem cell factor (100 ng/mL, PeproTech) or used for RNA extraction (Aurum™ Total RNA Mini Kit, Biorad).

Cell lines. The human cell lines THP1, SEM, K562, Kasumi-1 and murine cell line MLL-AF9 were a kind gift from Dr. Jürg Schwaller (Laboratory of childhood leukemia, Basel University, Switzerland). The human Nalm6 cell line was a kind gift from Prof. Noritaka Adachi (Yokohama City University, Yokohama, Japan). All generated cell lines tested negative for mycoplasma contamination

Growth curve analysis. To monitor the growth of all different cell lines, 50 000 cells/mL were plated six-fold in a 24-well plate. Every 24 hours, the number of cells per mL of 1 well was counted using trypan blue (Fisher Scientific) and TC20™ Automated Cell Counter from Biorad. Ln-transformed cell counts were analyzed using GraphPad Prism 8 to determine the doubling time.

Supplementary figures

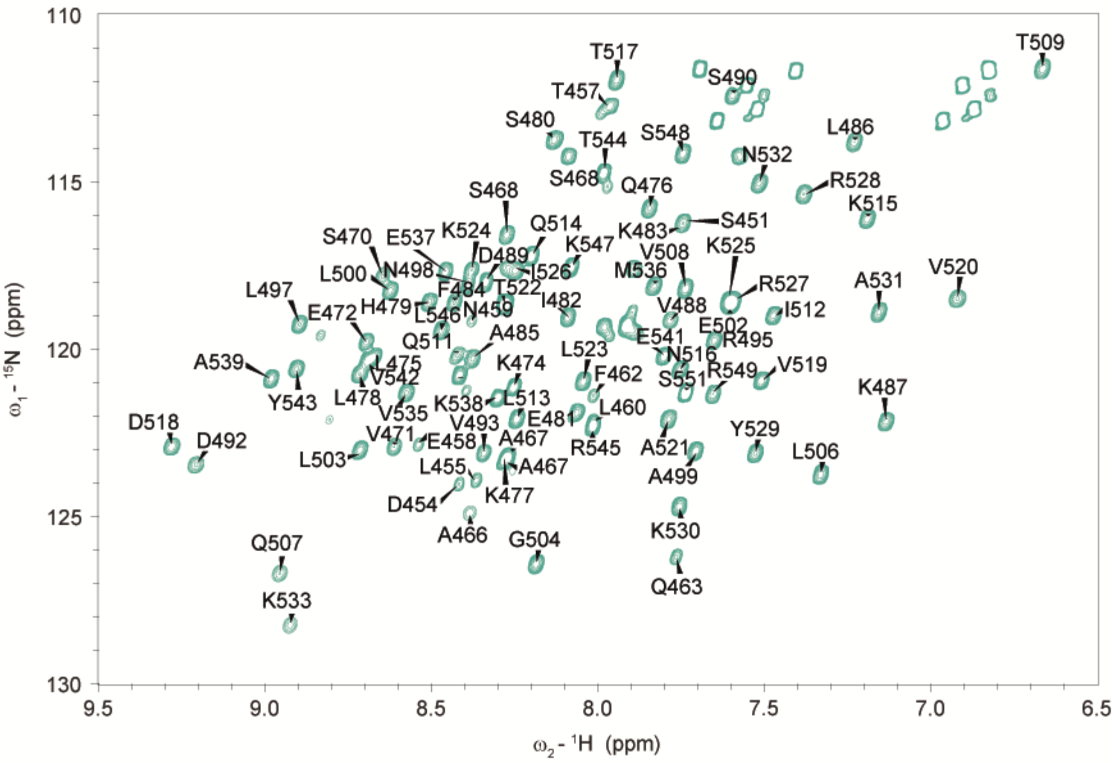

Supplementary figure 1: Assignment of the NMR 2D 15N/1H HSQC spectra of the 15N-labeled HRP-2 IBD.

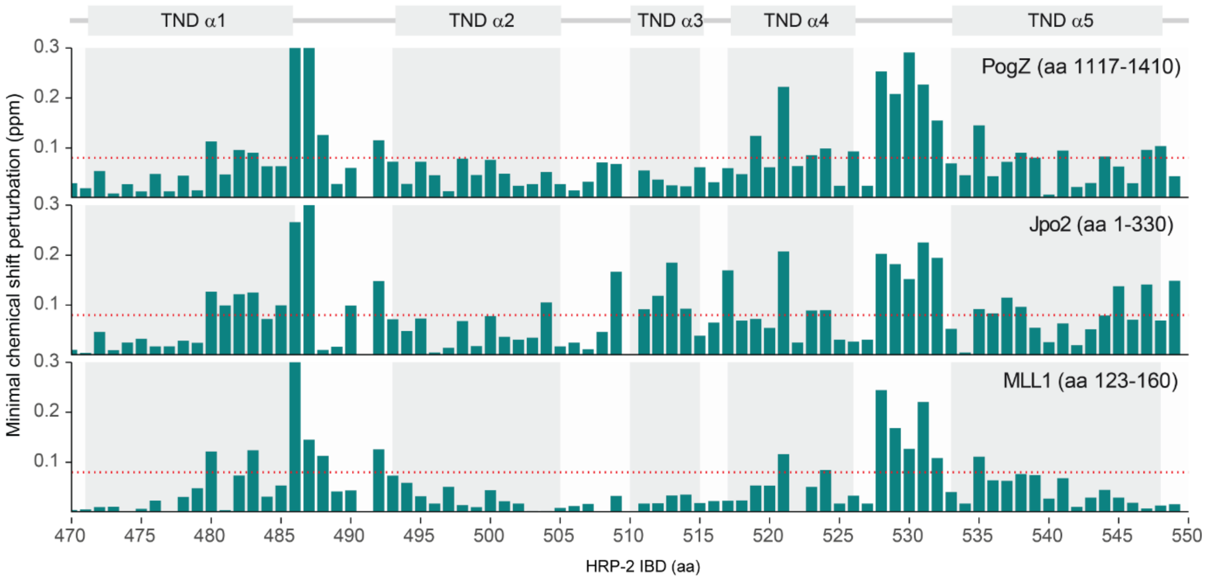

Supplementary figure 2: Amino acids perturbed by HRP-2 binding to JPO2 and PogZ are similar to the amino acids perturbed by MLL. HRP-2 IBD interacts with known LEDGF/p75 interacting partners that contain the conserved LEDGF/p75 IBD binding motif. Comparison of the minimal chemical shift perturbations (CSP) in HRP-2 IBD backbone amide signals in the presence of MLL, JPO2 and PogZ.

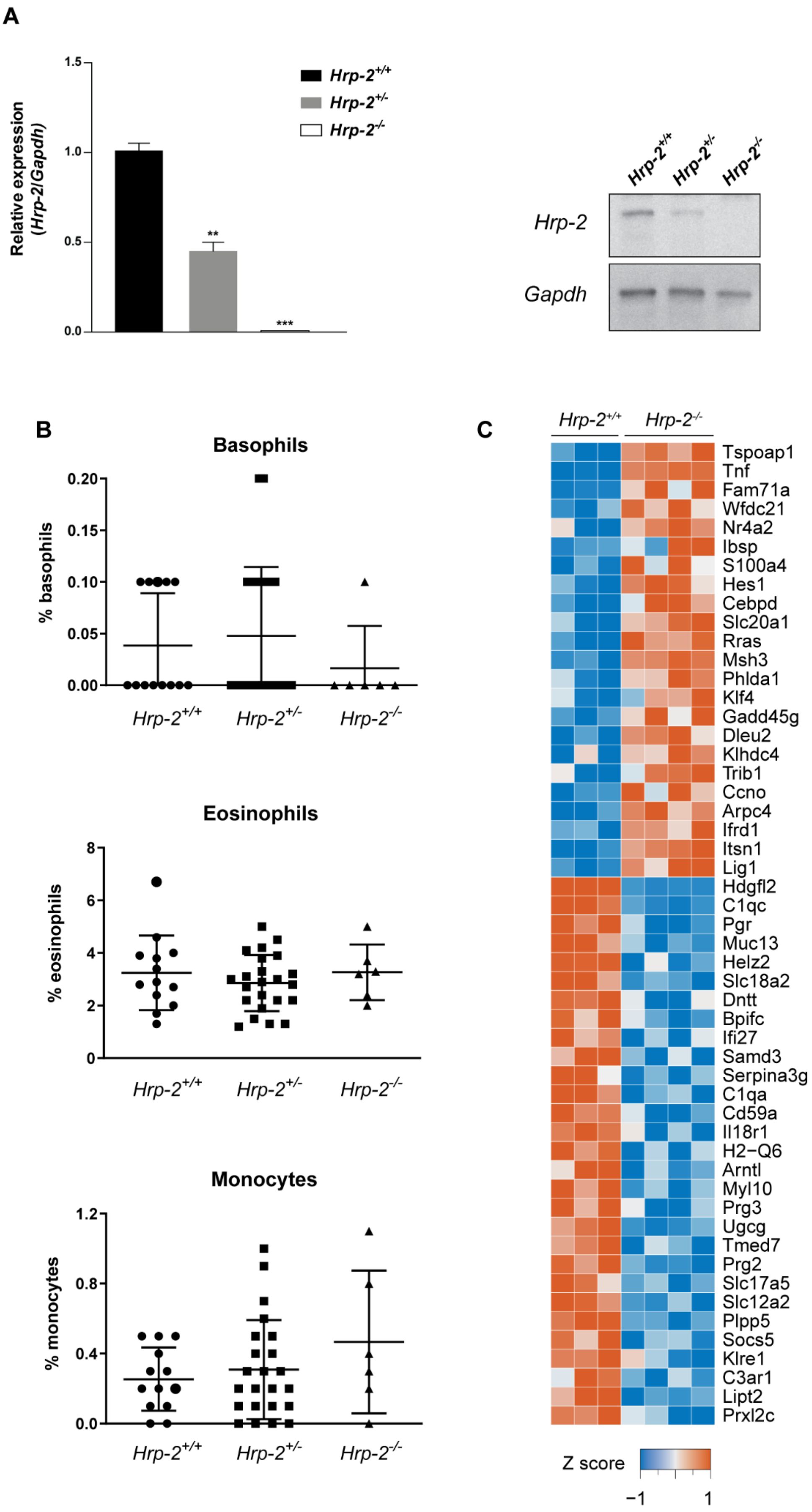
A
Relative expression (Hrp-2/Gapdh)
1.5
1.0
0.5
0.0
Hrp-2+/+
Hrp-2+/-
Hrp-2-/-
**
***
Hrp-2
Gapdh
B
Basophils
% basophils
0.20
0.15
0.10
0.05
0.00
Eosinophils
% eosinophils
8
6
4
2
0
Monocytes
% monocytes
1.2
0.8
0.4
0.0
C
Tspoap1
Tnf
Fam71a
Wfdc21
Nr4a2
Ibsp
S100a4
Hes1
Cebpd
Slc20a1
Rras
Msh3
Phlda1
Klf4
Gadd45g
Dleu2
Klhdc4
Trib1
Ccno
Arpc4
Ifrd1
Itsn1
Lig1
Hdgfl2
C1qc
Pgr
Muc13
Helz2
Slc18a2
Dntt
Bpifc
Ifi27
Samd3
Serpina3g
C1qa
Cd59a
Il18r1
H2−Q6
Arntl
Myl10
Prg3
Ugcg
Tmed7
Prg2
Slc17a5
Slc12a2
Plpp5
Socs5
Klre1
C3ar1
Lipt2
Prxl2c
Z score
−1
1

Supplementary figure 3: validation of HRP-2 mouse model. (A, left) qRT-PCR of lineage depleted bone marrow cells validating the complete HRP-2 depletion in *HRP-2* knockout (*HRP-2*$^{-/-}$) mice, compared to wild type (*HRP-2*$^{+/+}$) and heterozygous (*HRP-2*$^{+/-}$) animals. Expression levels were normalized to *Gapdh*. Error bars indicate standard deviations of triplicate measurements. Differences were determined using Student's t-test; **$p<0.01$, ***$p< 0.001$; (A, right) Western Blot of whole bone marrow cells to validate HRP-2 protein expression levels in *HRP-2* wild type (*HRP-2*$^{+/+}$), heterozygous (*HRP-2*$^{+/-}$) and knockout (*HRP-2*$^{-/-}$) animals. GAPDH was used as loading control. Details about the western blot analysis can be found in supplementary figure 8; (B) Comparison of differential white blood cell groups between wild type (*HRP-2*$^{+/+}$), heterozygous (*HRP-2*$^{+/-}$) and knockout (*HRP-2*$^{-/-}$) mice; (C) Heat map of differentially expressed genes detected by RNA-seq for *HRP-2*$^{-/-}$ lineage depleted bone marrow (lin^{-}) cells compared to *HRP-2*$^{+/+}$ lin^{-} cells. Color scale indicates the upregulated (red) and downregulation (blue) genes. Each row represents one animal.

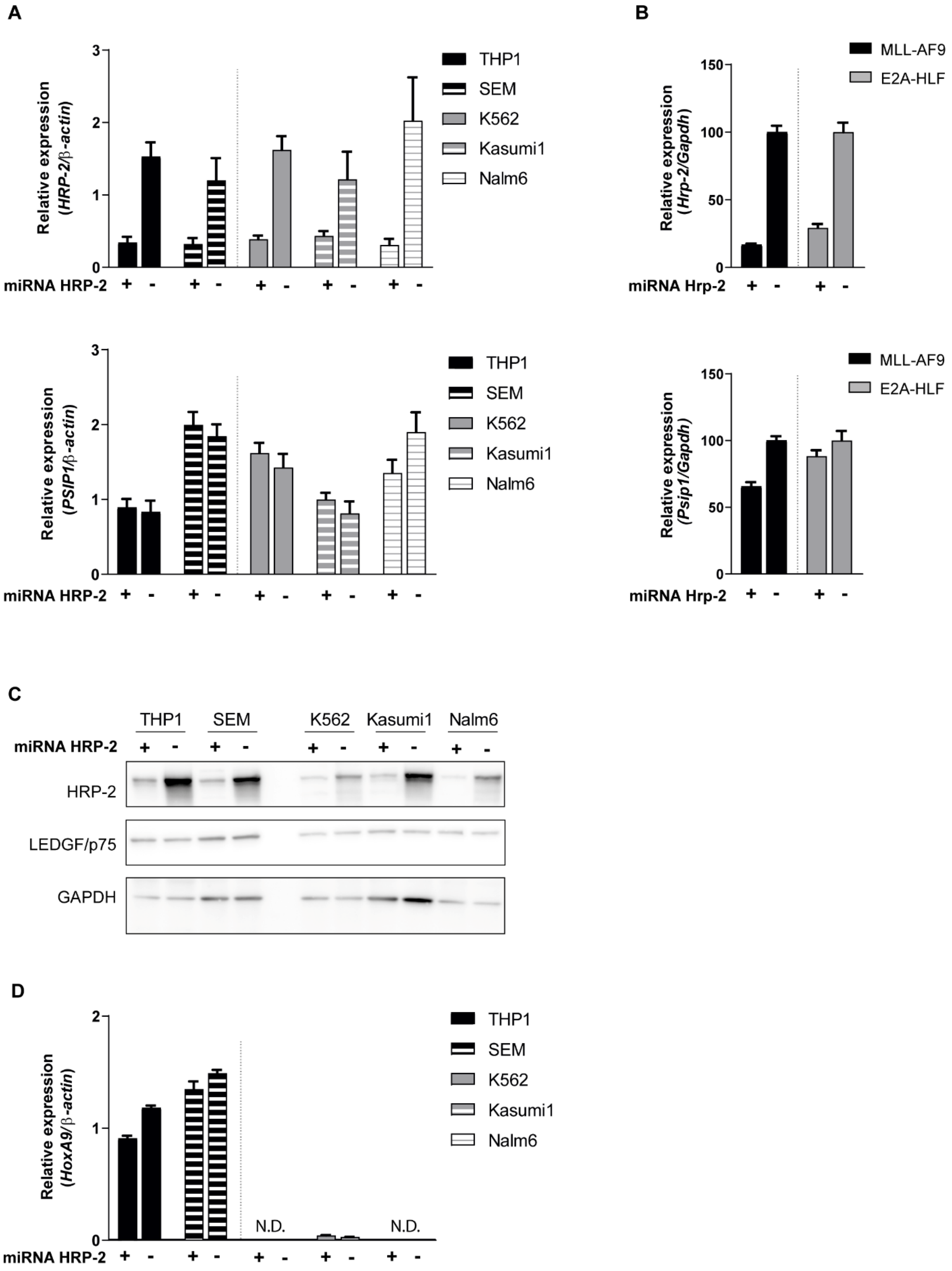
A
Relative expression (HRP-2/β-actin)
miRNA HRP-2 + - + - + - + - + -
THP1
SEM
K562
Kasumi1
Nalm6
Relative expression (PSIP1/β-actin)
B
Relative expression (Hrp-2/Gapdh)
Relative expression (Psip1/Gapdh)
miRNA Hrp-2 + - + -
MLL-AF9
E2A-HLF
C
THP1
SEM
K562
Kasumi1
Nalm6
miRNA HRP-2
HRP-2
LEDGF/p75
GAPDH
D
Relative expression (HoxA9/β-actin)
N.D.
N.D.

Supplementary figure 4. Validation of *HRP-2* knockdown in human and mouse cell lines. (A-B) mRNA levels of *HRP-2* (top) and *PSIP1* (LEDGF/p75, bottom) in whole cell lysates of the (A) human and (B) murine cell lines used. Expression levels were verified by qPCR and normalized to *β-actin* (human) or *Gapdh* (mouse). One representative experiment shown (n=3). Error bars indicate the standard deviation of three replicates; (C) Western blot analysis of HRP-2 and LEDGF/p75 protein expression levels after transduction with miRNA to knock down HRP-2 (miRNA HRP-2 +) or control (miRNA HRP-2 -). GAPDH was used as loading control. Details about the western blot analysis can be found in supplementary figure 8; (D) qPCR analysis of HOXa9 expression levels in human cell lines, normalized to *β-actin*. One representative experiment shown (n=3). Error bars indicate the standard deviation of duplicates. N.D. = not detectable

A

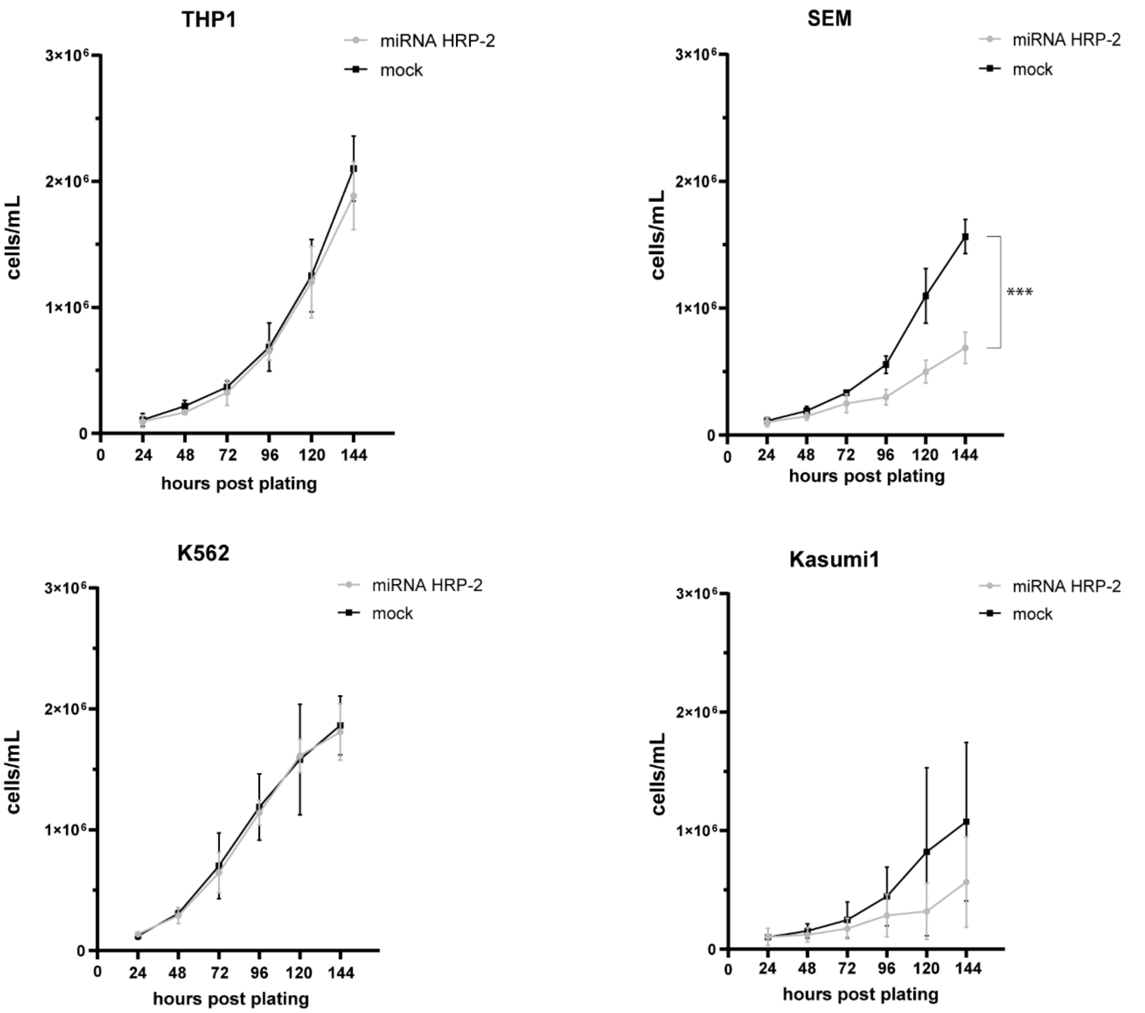

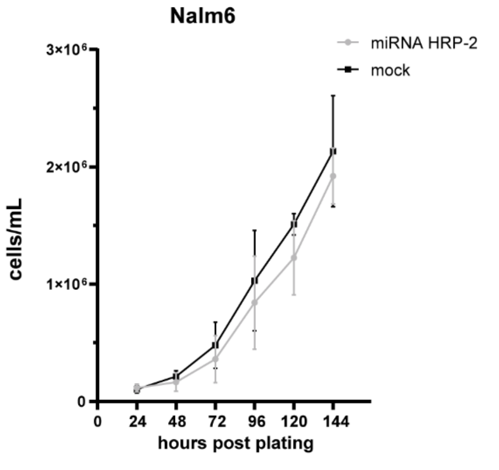

B

Estimated doubling time		
	miRNA HRP-2	mock
THP1	26 hours 52 min	27 hours 52 min
SEM	43 hours 12 min	30 hours 46 min
K562	31 hours 55 min	31 hours 45 min
Kasumi1	50 hours 49 min	35 hours 58 min
Nalm6	27 hours 1 min	26 hours 57 min

Supplementary figure 5. HRP-2 depletion variably affects growth of human cell lines. (A) Growth of cultured cells was monitored for six consecutive days by counting the number of cells per mL culture. Error bars indicate the standard deviation of three independent replicates. Differences at the end point were determined using Mann-Whitney U test; ***$p < 0.003$; (B) Inserted table comparing doubling time between HRP-2 depleted (miRNA HRP-2) and control (mock) cells lines from the represented graphs.

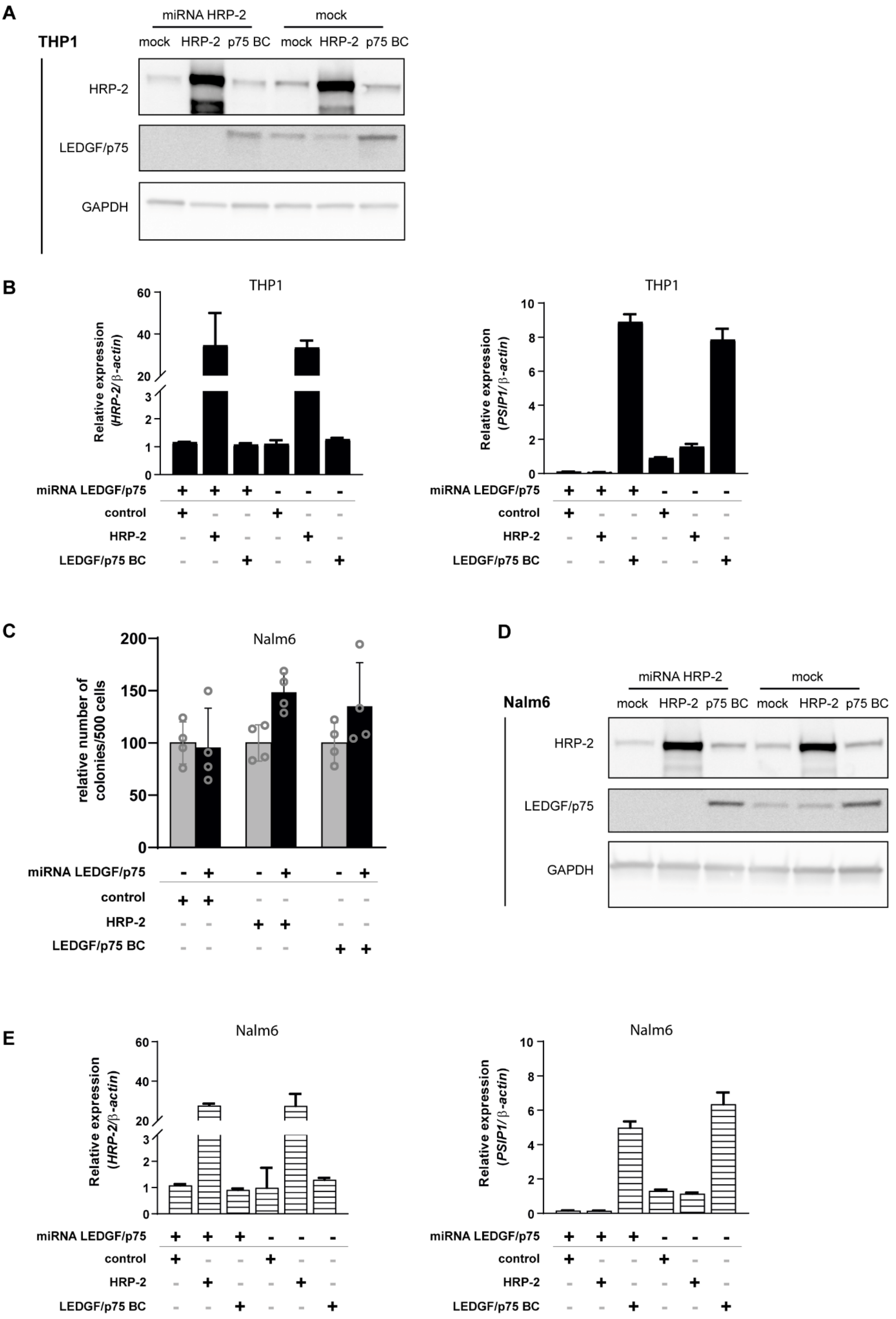
A
THP1
miRNA HRP-2
mock
mock HRP-2 p75 BC mock HRP-2 p75 BC
HRP-2
LEDGF/p75
GAPDH
B
THP1
Relative expression (HRP-2/β-actin)
Relative expression (PSIP1/β-actin)
miRNA LEDGF/p75
control
HRP-2
LEDGF/p75 BC
C
Nalm6
relative number of colonies/500 cells
D
Nalm6
E
Nalm6

Supplementary figure 6. Validation of double transduced THP1 and Nalm6 cells used for CFU experiment. (A-B) Whole cell lysates of THP1 cells either transduced with an LEDGF/p75-miRNA to knockdown LEDGF/p75 (+) or control (-) and complemented with HRP-2, a miRNA-resistant LEDGF/p75 or mock vector (control) were validated by (A) Western Blot using specific antibodies and (B) qPCR analysis for *HRP-2* and *PSIP1* (LEDGF/p75). One representative experiment is shown (n=3). Error bars in B represent standard deviation of duplicates. Expression levels were normalized to β-*actin*; (C) Colony formation of stably transduced Nalm6 cell lines with an LEDGF/p75-miRNA to knockdown LEDGF/p75 (+) or control (-) and complemented with HRP-2, a miRNA-resistant LEDGF/p75 or mock vector (control). Error bars indicate standard deviation of four replicates. Replicates are indicated by open circles; (D-E) Whole cell lysates of all stably transduced Nalm6 cells were validated by (D) Western Blot using specific antibodies and (E) qPCR analysis for *HRP-2* and *PSIP1* (LEDGF/p75). One representative experiment is shown (n=3). Error bars in E represent standard deviation of duplicates. Expression levels were normalized to β-*actin*. Details about the western blot analysis can be found in supplementary figure 8.

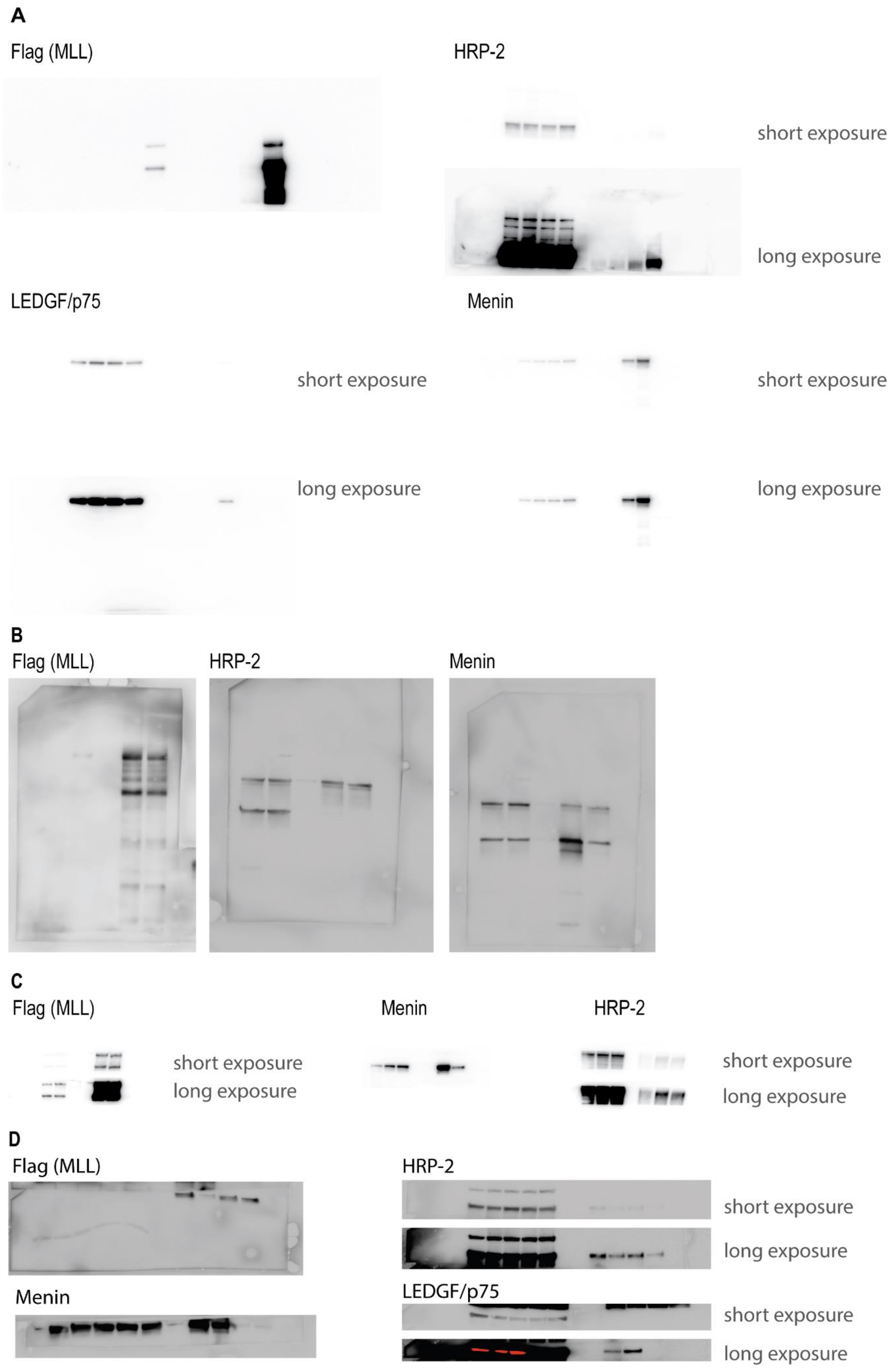
A
Flag (MLL)
HRP-2
short exposure
long exposure
LEDGF/p75
short exposure
long exposure
Menin
short exposure
long exposure
B
Flag (MLL)
HRP-2
Menin
C
Flag (MLL)
short exposure
long exposure
Menin
HRP-2
short exposure
long exposure
D
Flag (MLL)
HRP-2
short exposure
long exposure
Menin
LEDGF/p75
short exposure
long exposure

Supplementary figure 7. Original blots from co-immunoprecipitation experiments. (A) blot corresponding figure 1B. All samples were run on the same blot and the blot was cut horizontally to visualize multiple proteins. After Flag and LEDGF/p75 detection, the blots were stripped and used for detection of HRP-2 and menin, respectively. Input (left) and IP (right) were separated by a protein marker. As indicated, longer exposure was needed to visualize HRP-2 and LEDGF/p75 IP-bands. The samples were loaded in the same order as indicated in the cropped figure. Antibody detection was performed using Amersham ImageQuant 800 (GE Healthcare); (B) blot corresponding figure 1C. After HRP-2 detection, the same blot was stripped and used for detection of menin. Detection of Flag (MLL) was performed on a different blot, coming from the same experimental samples. Samples were consecutively loaded in the same order as indicated in the cropped figure. Blots were cut horizontally to visualize multiple proteins on one blot. Antibody detection was performed using LAS 3000 mini (GE Healthcare); (C) blot corresponding figure 1D. All samples were run on the same blot and the blot was cut horizontally to visualize different proteins separately. To visualize the Flag (MLL) input and HRP-2 IP signals, blots were exposed longer. Input (left) and IP (right) were separated by a protein marker. The samples were loaded in the same order as indicated in the cropped figure. Antibody detection was performed using Amersham ImageQuant 800 (GE Healthcare); (D) blot corresponding figure 1E. To visualize the HRP-2 and LEDGF/p75 IP signals, blots were exposed longer. HRP-2 and LEDGF/p75 were run and analyzed on the same blot. Here, input and IP were separated by a protein marker. Samples were reloaded to detect Flag (MLL) and menin. Here, samples were loaded consecutively. Antibody detection was performed using LAS 3000 mini (GE Healthcare).

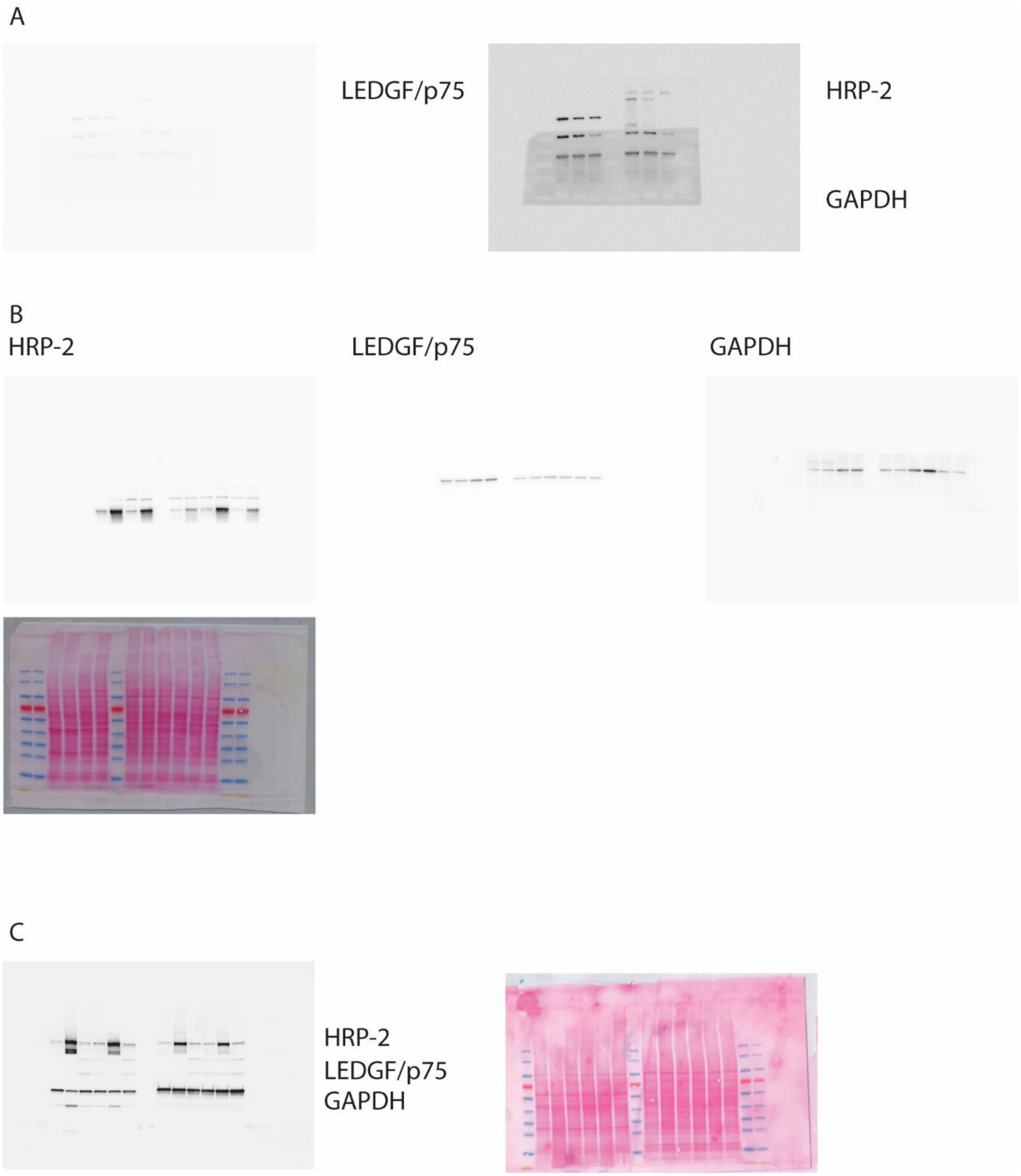

Supplementary Figure 8. Original blots to validate HRP-2 and LEDGF/p75 expression levels. (A) blot corresponding supplementary figure 3A. left blot, unadjusted blot with the three HRP-2 genotypes. Right blot, complete blot adjusted by brightness/contrast function imageJ. Wild type – heterozygous – knockout primary cells were loaded two times, separated by protein marker. Antibody detection of LEDGF/p75 (upper left), HRP-2 (upper right) and GAPDH (below) as loading control. Antibody detection was performed using LAS 3000 mini (GE Healthcare); (B) blots corresponding supplementary figure 4C. MLL-rearranged samples (THP1, SEM) were separated from MLL wt cell lines (K562, Kasumi1, Nalm6) with a protein marker. For every cell line, the HRP-2 KD condition was loaded before the control condition as indicated in the cropped figure. To analyze HRP-2, LEDGF/p75 and GAPDH on the same blot, the blot was cut horizontally at the 100 kDa and 55 kDa marker. Antibody detection

was performed using Amersham ImageQuant 800 (GE Healthcare). Besides GAPDH, a ponceau stain was performed to assess equal loading; (C) blots corresponding supplementary figure 6A and 6D. THP1 (left) and Nalm6 (right) samples were run on the same blot and separated by a protein marker. The samples were loaded as indicated in the cropped figures. To analyze HRP-2, LEDGF/p75 and GAPDH on the same blot, the blot was cut horizontally at the 100 kDa and 55 kDa marker. Antibody detection was performed using Amersham ImageQuant 800 (GE Healthcare). Besides GAPDH, a ponceau stain was performed to assess equal loading.

Supplementary table 1: Statistics for the final water-refined sets of structures

**Non-redundant distance and angle constrains**	
Total number of NOE constraints	2528
Short-range NOEs	
Intra-residue (i = j)	501
Sequential (| i - j | = 1)	610
Medium-range NOEs (1 < | i - j | < 5)	814
Long-range NOEs (| i - j | ≥ 5)	602
Torsion angles	144
Total number of restricting constraints	2672
Total restricting constraints per restrained residue	32.6
**Residual constraint violations**	
Distance violations per structure	
0.1 – 0.2 Å	11.2
0.2 – 0.5 Å	0.5
> 0.5 Å	0
r.m.s. of distance violation per constraint	0.02 Å
Maximum distance violation	0.28 Å
Dihedral angle viol. per structure	
1 – 10 °	4.0
> 10 °	0
r.m.s. of dihedral violations per constraint	0.57°
Maximum dihedral angle viol.	5.0°
**Ramachandran plot summary**	
Most favored regions	92.6 %
Additionally allowed regions	7.4 %
Generously allowed regions	0.0 %
Disallowed regions	0.0 %
**r.m.s.d. to the mean structure**	*all/ordered*[1]
All backbone atoms	0.5/0.1 Å
All heavy atoms	0.6/0.6 Å
PDB entry	6T3I
BMRB accession code	34442

[1]Residues with sum of phi and psi order parameters > 1.8

References

1. Schrijvers, R.; De Rijck, J.; Demeulemeester, J.; Adachi, N.; Vets, S.; Ronen, K.; Christ, F.; Bushman, F.D.; Debyser, Z.; Gijsbers, R. LEDGF/p75-independent HIV-1 replication demonstrates a role for HRP-2 and remains sensitive to inhibition by LEDGINs. *PLoS Pathog.* **2012**, *8*, doi:10.1371/journal.ppat.1002558.

2. Méreau, H.; De Rijck, J.; Čermáková, K.; Kutz, A.; Juge, S.; Demeulemeester, J.; Gijsbers, R.; Christ, F.; Debyser, Z.; Schwaller, J. Impairing MLL-fusion gene-mediated transformation by dissecting critical interactions with the lens epithelium-derived growth factor (LEDGF/p75). *Leukemia* **2013**, *27*, 1245–1253, doi:10.1038/leu.2013.10.

3. Cermakova, K.; Tesina, P.; Demeulemeester, J.; El Ashkar, S.; Méreau, H.; Schwaller, J.; \vRezáčová, P.; Veverka, V.; De Rijck, J. Validation and structural characterization of the LEDGF/p75-MLL interface as a new target for the treatment of MLL-dependent leukemia. *Cancer Res.* **2014**, *74*, 5139–5151.

4. Tesina, P.; Čermáková, K.; Hořejší, M.; Procházková, K.; Fábry, M.; Sharma, S.; Christ, F.; Demeulemeester, J.; Debyser, Z.; Rijck, J. De; et al. Multiple cellular proteins interact with LEDGF/p75 through a conserved unstructured consensus motif. *Nat. Commun.* **2015**, *6*, 7968, doi:10.1038/ncomms8968.
